# Optimization of physical factors affecting the production of thermo-stable organic solvent-tolerant protease from a newly isolated halo tolerant *Bacillus subtilis *strain Rand

**DOI:** 10.1186/1475-2859-8-20

**Published:** 2009-04-09

**Authors:** Randa A Abusham, Raja Noor Zaliha RA Rahman, Abu Bakar Salleh, Mahiran Basri

**Affiliations:** 1 Enzyme and Microbial Technology Research Group, Faculty of Biotechnology and Biomolecular Sciences, Universiti Putra Malaysia, 43400 UPM Serdang, Selangor, Malaysia; 2 Enzyme and Microbial Technology Research Group, Faculty of Science, Universiti Putra Malaysia, 43400 UPM Serdang, Selangor, Malaysia

## Abstract

**Background:**

Many researchers have reported on the optimization of protease production; nevertheless, only a few have reported on the optimization of the production of organic solvent-tolerant proteases. Ironically, none has reported on thermostable organic solvent-tolerant protease to date. The aim of this study was to isolate the thermostable organic solvent-tolerant protease and identify the culture conditions which support its production. The bacteria of genus *Bacillus *are active producers of extra-cellular proteases, and the thermostability of enzyme production by *Bacillus *species has been well-studied by a number of researchers. In the present study, the *Bacillus subtilis *strain Rand was isolated from the contaminated soil found in Port Dickson, Malaysia.

**Results:**

A thermostable organic solvent-tolerant protease producer had been identified as *Bacillus subtilis *strain Rand, based on the 16S rRNA analysis conducted, as well as the morphological characteristics and biochemical properties. The production of the thermostable organic solvent-tolerant protease was optimized by varying various physical culture conditions. Inoculation with 5.0% (v/v) of (AB_600 _= 0.5) inoculum size, in a culture medium (pH 7.0) and incubated for 24 h at 37°C with 200 rpm shaking, was the best culture condition which resulted in the maximum growth and production of protease (444.7 U/ml; 4042.4 U/mg). The Rand protease was not only stable in the presence of organic solvents, but it also exhibited a higher activity than in the absence of organic solvent, except for pyridine which inhibited the protease activity. The enzyme retained 100, 99 and 80% of its initial activity, after the heat treatment for 30 min at 50, 55, and 60°C, respectively.

**Conclusion:**

Strain Rand has been found to be able to secrete extra-cellular thermostable organic solvent-tolerant protease into the culture medium. The protease exhibited a remarkable stability towards temperature and organic solvent. This unique property makes it attractive and useful to be used in industrial applications.

## Background

Currently, enzymes have attracted the world attention due to their wide range of industrial applications in many fields, including organic synthesis, clinical analysis, pharmaceuticals, detergents, food production and fermentation. Enzymes are gradually replacing the use of harsh chemicals in various industrial processes [1]. Proteases are one of the most important groups of industrial enzymes and they account for nearly 60% of the total enzyme sale [2-4].

In industrial applications, with thermopiles and thermostable enzymes, the isolation of enzymes is dominating over micro-organisms [5]. Bacterial proteases, especially from *Bacillus *sp., are the most widely exploited industrial enzymes and among the bacteria, *Bacillus *sp., are producers of extra-cellular proteases [6].

The industrial use of proteases, in detergents and in leather processing, requires that the enzymes be stable at higher temperatures. Thermostable proteases are advantageous in some applications because of the higher processing temperatures which can be employed, resulting in much faster reaction rates, increasing the solubility of non-gaseous reactants and products, and reducing the incidence of microbial contamination by mesophilic organisms [7].

Thermophilic enzymes are potentially applicable in a wide range of industrial processes, particularly and mainly due to their denaturant tolerance and extraordinary operational stability at high temperatures. Such enzymes are used in chemical, food, pharmaceutical, paper, textile and other industries [5,8,9].

Enzymatic conversions in non-aqueous media have been shown to possess many potential industrial applications. The areas of application vary widely from food additives, flavours and fragrances to pharmaceuticals, pesticides and specialty polymers [10]. Enzymes, which are stable and active in non-aqueous media, are in large demand for their increasing application in organic synthesis [11]. The use of proteases in peptide synthesis is limited by the specificity and the instability of the enzymes in the presence of organic solvents, since reactions occurred in organic media. However, little attention has been given to the study of organic solvent-stable protease [12].

Each organism or strain has its own special conditions for the maximum enzyme production [13]. The general rules for the optimization of microbial protease production are affected by various physical factors which include pH, cultivation temperature, shaking condition and aeration. These factors are important to promote, stimulate, enhance and optimize the production of proteases [14]. However, cultivation conditions are essential in a successful production of an enzyme, while optimization parameters, such as pH and temperature, are important in developing this cultivation process [15].

In this study, the effects of physical factors on the production of a thermostable organic solvent-tolerant protease, from *Bacillus subtilis *strain Rand, were identified and investigated.

## Results and discussion

### Screening and isolation of bacteria

Contamination and hot surrounding area may provide a good environment for the growth of micro-organisms producing thermostable, organic solvent-tolerant proteases. Several samples were obtained from a car service workshop located in Port Dickson, and hot spring water from Batang Kali and Selayang, Malaysia. The temperatures were between 45 to 90°C when the sample was collected. From a comprehensive screening on the Skim Milk Agar (SMA) plate, ten isolates (L1, L2, BK, BK1, BK2, PD, PD1, PD2, PD and Rand) showed positive results by forming zone of lyses around the colonies on the SMA. All these isolates were found to be able to produce protease (data not shown). Among the ten isolates, Rand was detected to have the highest protease activity (34.9 U/ml), and was selected for further study.

### Identification of isolate Rand

#### 16S rDNA analysis

**Figure 1:**
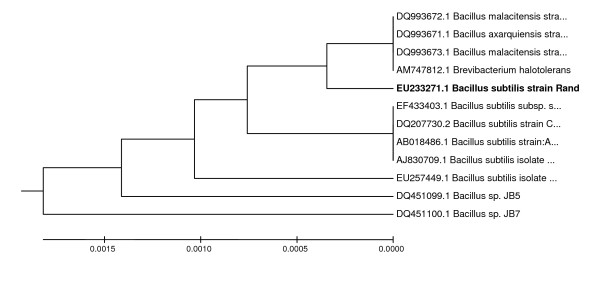
**Phylogenetic position of strain Rand with other bacteria**. The members of bacteria used include *Bacillus malacitensis *CECT 5687; *Bacillus axarquiensis *LMG 22476; *Bacillus malacintesis *LMG 22477; *Brevibacterium halotolerans*;*Bacillus subtilis *subsp. spizizenii BCRC 10447;*Bacillus subtilis *CCM 1999; *Bacillus subtilis *AU30; *Bacillus subtilis *isolate KCM-RG5; *Bacillus subtilis *isolate C10-1. Phylogenetic tree was inferred by using the neighbour-joining methods. The software package MEGA 4 was used for analysis.

The primers are highly conserved among prokaryotes and found to amplify the whole region of the rRNA gene, which is 1500 bp. The PCR product sequencing was done by the First BASE Laboratories Sdn Bhd (Shah Alam, Selangor, Malaysia). The DNA homology search on the GenBank database  was performed. A phylogenetic tree was constructed based on the comparison of the 16S rDNA sequence of this isolate and other strain of *Bacillus*. All the sequences were aligned with CLUSTALW from Biology Workbench database at [16]. 16S rDNA sequences of other *Bacillus *were obtained from the GenBank database . The results gathered from the 16S rDNA analyses show that *Bacillus subtilis *strain Rand is very close to *Brevibacterium halotolerans*, *Bacillus malacintesis *strain LMG 22477, *Bacillus malacitensis *strain CECT 5687 and *Bacillus axarquiensis *strain LMG 22476 (Figure 1). The partial sequencing of the 16S rDNA shows a 99.6% similarity to different strains of *Bacillus subtilis*. In addition, the analysis of the cellular fatty acids also shows a good correspondence to the profile of the *Bacillus subtilis *group.

Strain Rand is an aerobic, rod-shaped, with 0.7–0.8 μm in width and 2.5–3.0 μm in length gram positive bacteria. The biochemical, morphological and physiological properties of strain Rand are listed in Table 1.

**Table 1:** Morphological and biochemical characteristics of *Bacillus subtilis *strain Rand

**Characteristics**	**Isolate Rand results**
Rods	+
Width μm	0.7–0.8
Length μm	2.5–3.0
	
Aminopeptidase test	-
KOH test	-
Oxidase	+
Catalase	+
	
Gram stain	+
	
Spores	+
Sporangium swollen	-
	
Anaerobic growth	-
VP reaction	+
pH in VP broth	5.5
	
Growth positive at	50°C
Growth negative at	55°C
	
Growth in medium pH5.7	+
NaCl 2%	+
5%	+
7%	+
10%	+
	
Acid form	
D-glucose	+
L-arabinose	+
D-xylose	+
D-mannitol	+
D-frucrose	+
	
Use of citrate	+
propionate	-
	
NO_2 _from NO_3_	+
Indol reaction	-
Phenylalanine deaminase	-
Arginine dihydrolase	-
	
Hydrolysis of Starch	+
Gelatin	+
Casein	+
Tween 80	+

According to the 16S rDNA analysis, the biochemical results and morphological properties of the bacterium were identified as *Bacillus subtilis *strain Rand.

### Organic solvent-stability of crude enzyme

Enzymes are usually inactivated or denaturated in the presence of organic solvents [12]. The effects of different organic solvents on protease stability were studied. The relative activity, which remained after 30 min of incubation in 25% (v/v) of organic solvent, is shown in Table 2. The activity of the enzyme, without any solvent (control), was taken as 100%. Rand protease showed a remarkable stability in the presence of all of the solvents, except pyridine (log *P *0.71) as shown in Table 2. The remaining activity of the Rand protease was found to be 104, 197, 130, 134, 146, 209, 151, 152 and 151% in the presence of butanol, benzene, toluene, *p*-xylene, *n*-hexane, *n*-decane, *n*-dodacane, *n*-tetradecane and *n*-hexadecane, respectively (data not shown). This level of stability, towards hydrophobic and hydrophilic solvents, is unique. The remaining activities of alkaliphilic protease, from *Bacillus subtilis *TKU07, were 65% and 90% in the presence of only 20% (v/v) of butanol and toluene [17]. Gupta and Khare reported that crude *P. aeruginosa *PseA protease showed a remarkable stability in the presence of most solvents, having the logarithm of the partition coefficient (log *P*) above 2.0, but was less stable in the presence of hydrophilic solvents [11]. The stability of the *Pseudomonas aeruginosa *protease, in the presence of organic solvents (of which the values of the log *P *were equal to or more than 3.2), was almost the same as the one found in the absence of organic solvents [18]. Purified protease, from *Pseudomonas aeruginosa *PseA strain, was found to be stable in the presence of a range of organic solvents, but was detected to be less stable in benzene and isooctane [19]. In another study, the protease from *Pseudomonas aeruginosa *stain K was activated when compared to the control, in the presence of 25% organic solvents, with the Log *P *values exceeding 4.0; however, in the presence of 25% organic solvents with the Log *P *values below 4.0, the stability of the protease was lesser after 30 min of incubation [20]. Slightly over 20% of the activity remained [21] in the crude protease derived from *Pseudomonas aeruginosa *san-ai, in the presence of butanol, chloroform, and hexane. However, the results in this study showed that the protease, from the Rand strain, was not only stable in the presence of various organic solvents (with the Log *P *values equalled to or more than 2.0), but also in the presence of some organic solvents with the Log P values below 2.0. These results indicated that this protease might be a novel solvent-stable protease.

**Table 2:** Effect of organic solvents on Rand protease stability

Organic solvents	Log P *	Protease activity (U/ml)	
		0 min	30 min
pyridine	0.71	22.66	0.0
butanol	0.80	23	38.3
benzene	2.0	61.9	72.7
toluene	2.5	45.7	47.9
*p*-xylene	3.1	54.4	49.6
*n*-hexane	3.5	47.22	53.9
*n*-decane	5.6	53.4	77.4
*n*-dodacane	6.0	65	55.7
*n*-tetradecane	7.6	44.7	56.4
*n*-hexadecane	8.8	61.9	56
None		37	37

* Adopted from Laane *et al*. [22]

Note: 25% (v/v) of organic solvents were added to the cell-free supernatant and incubated at 50°C with shaking 150 rpm for 30 min.

### Thermostability of crude enzyme

**Figure 2:**
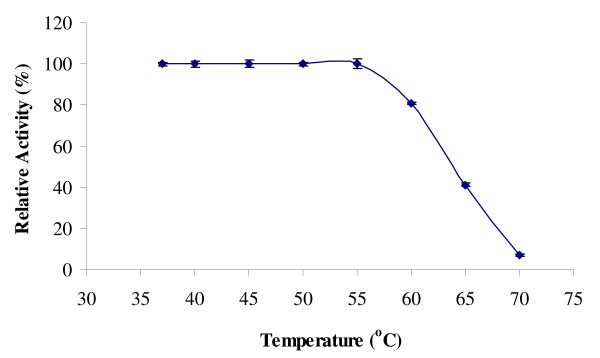
**Effect of Temperature on Protease Stability**. The crude enzyme was incubated at different temperatures (37–70°C) for 30 min with shaking 150 rpm. Protease activity at 37°C was considered as 100%.

Another remarkable feature of the Rand protease is its stability in high temperatures. To study the stability of enzyme at different temperatures, crude enzyme was pre-incubated at different temperatures ranging from 37 to 70°C for 30 min, rapidly cooled, and the protease activities were measured by the standard assay procedure. The protease appeared to be stable and was found to be able to retain its full activity after 30 min of incubation in the temperature ranging from 37 to 55°C (Figure 2). The crude enzyme retained 80% activity (0.086 mg/ml), even after the heat treatment at 60°C for 30 min. A reduction in the enzyme activity was observed at the temperature values above 60°C.

The Rand protease is more thermostable than other organic solvent tolerant proteases. Ghorbel *et al*. isolated a protease from *Bacillus cereus *BG1 which retained 89.5 of its original activity, after 15-min incubation at 55°C, in the presence of 2 mM Ca^2+^; meanwhile, no activity was detected in the absence of Ca^2+ ^[12]. An organic solvent-stable protease from *Pseudomonas aeruginosa *PST-01 was reported to be stable at the temperature below 50°C [23]. A solvent stable protease, from *Pseudomonas aeruginosa *PseA retained 80% of its initial activity after heating, for 30 min at 55°C [11]. In particular, TKU004 metalloprotease had 10% of its activity retained at 60°C, but was completely inactivated at 70°C [24]. The Rand protease displayed a greater stability at higher temperatures, and thus was suitable to be used in industrial and biotechnological applications.

### The effects of temperature on the production of protease

**Figure 3:**
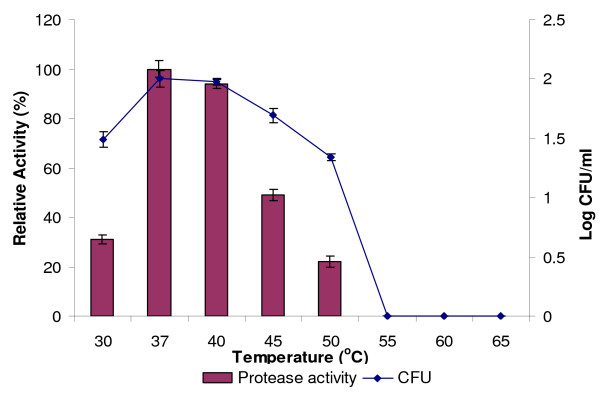
**Effect of temperature on protease production**. Culture media were incubated at 30, 37, 40, 45, 50, 55, 60 and 65°C with shaking at 150 rpm for 24 h. Protease activity at 37°C was considered as 100%.

Temperature is a critical parameter which needs to be controlled and this is usually varied from organism to another [13]. The optimum temperature for the production of protease and bacterial growth was investigated from 30°C to 65°C. In shaken cultures, 37°C was found to the optimum temperature for both protease production and bacterial growth (Figure 3). The incubation at 30, 40, 45 and 50°C was found to decrease the production of protease, and no protease activity was detected at 55, 60 and 65°C. The optimum temperature for crude protease, produced from *B*. *subtilis *strain 38, was 47°C [25]. The optimum temperature for the protease produced by *Bacillus *sp. MIG was found to be 30°C [26]. Meanwhile, the optimum temperature for the production of protease by *Bacillus *sp SMIA-2 was 60°C [7]. The optimum temperature for the protease produced by *Bacillus licheniformis *was 50°C [27]. The studies by Frankena *et al*. [28] showed that there was a link between enzyme synthesis and energy metabolism in bacteria, and this was controlled by the temperature and oxygen uptake. As for the extra-cellular enzymes, temperature was found to influence their secretion, possibly by changing the physical properties of the cell membrane [14]. On the other hand, a lower growth of Rand strain at high temperatures could be due to the lack of dissolved oxygen in the medium, which resulted to a low protease activity. It is a well-known fact that protein conformation changes or degraded at higher temperatures, and hence, causes a decrease in the protease activity [29].

### The effects of pH on the production of protease

**Figure 4:**
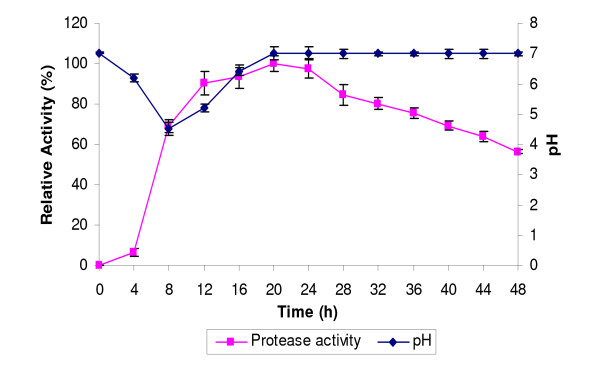
**Time course of protease activity and pH of the medium**. Culture media were incubated at 37°C with shaking at 150 rpm for 48 h. Samples were taken at 4 h intervals to determine the protease activity and the pH level.

The pH of the medium started to decrease after 4 h to 4.5 after 8 h of growth (Figure 4). The increased acidity was due to the production of acids during the bacterial growth. After that, the pH was slightly increased to 5.2 at 12 h, and to 6.4 at 16 h. It turned neutral at 20 h and remained constant up to 48 h. The rise in the pH after 8 h of incubation could be due to utilization of organic acids or the production of alkaline compounds.

**Figure 5:**
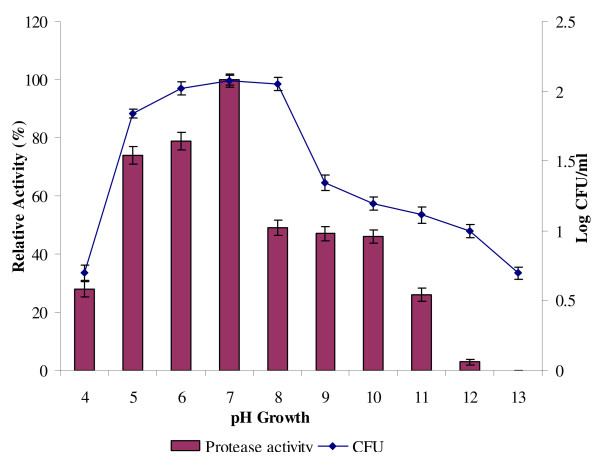
**Effect of pH on protease production**. Bacterial cultures were adjusted to pH 4, 5.0, 6.0, 7.0, 8.0, 9.0, 10.0, 11.0, 12.0, 13.0 and incubated at 37°C with 150 rpm for 24 h. Protease activity at pH 7 was considered as 100%.

Extra-cellular protease was detected over a broad pH range (pH 4.0 to 9.0), with the optimum production of protease and bacterial growth exhibited at pH 7.0 (Figure 5). The bacterial growth and production of protease in an acidic medium at pH 6.0 were higher as compared to that in alkaline at pH 8.0. However, at an extreme acidity of pH 4.0, the production of protease was found to be greatly reduced. The optimum pH for the production of protease determined in this study is in agreement with the optimum pH for the protease from *Bacillus sp*. MIG [26]. The crude protease enzyme, produced from *B*. *subtilis *strain 38, had the optimal pH at 6.5 [25]. Malathu *et al*. reported an extra-cellular protease from a novel bacterial isolate showing the maximum activity at pH 7.5 [1]. Meanwhile, Nascimento and Martins reported an optimum pH of 8.0 for a protease derived from therophilic *Bacillus *sp strains SMIA-2 [7].

Moon and Parulekar [30] reported that the pH of culture has been shown to strongly affect many enzymatic processes and transportation of various components across the cell membrane.

### The effect of agitation rate on the production of protease

Micro-organisms vary in their oxygen requirements. In particular, oxygen acts as a terminal electron acceptor for oxidative reactions to provide energy for cellular activities. The variation in the agitation speed has been found to influence the extent of mixing in the shake flasks or the bioreactor, and also affect the nutrient availability [7].

**Figure 6:**
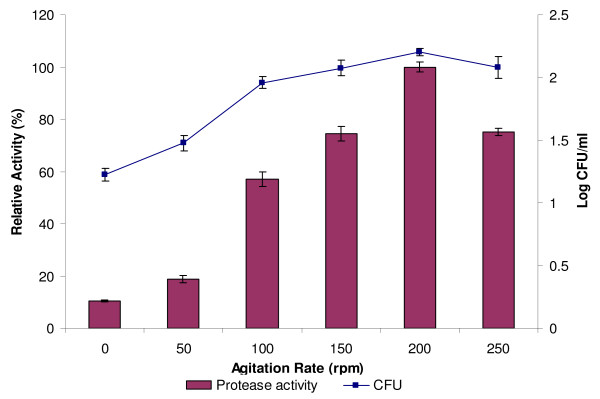
**Effect of agitation rate on protease production**. Culture media were incubated at 37°C with different shaking rates (0, 50, 100, 150, 200 and 250 rpm) for 24 h. Protease activity at 200 rpm was considered as 100%.

The effects of agitation rate on the production of protease and bacterial growth were investigated. Figure 6 shows that the highest protease production and bacteria growth were obtained when agitated at 200 rpm. At this speed, the aeration of the culture medium was increased, and this further led to a sufficient supply of dissolved oxygen in the media [13]. Although the production of protease was found to decrease when shaken at 250 rpm, the static condition almost inhibited its production.

As a conclusion, higher agitation rates could increase the oxygen pressure of the system but did not bring about the increase in production, probably because at a high agitation rate, the structure of enzyme would be altered [31]. However, lowering the aeration rate could cause a drastic reduction in the protease yields [24]. This indicates that a reduction in oxygen supply is an important limiting factor for growth as well as protease synthesis [7].

### The effects of inoculum size on the production of protease

The finite volume of a culture medium means that it can only contain limited nutrients for the micro-organism. Furthermore, the consumption of the nutrients is largely dependent on the population of bacteria. To ensure a high production of enzyme in the limited volume of medium, the bacterial inoculum size should therefore be controlled.

**Figure 7:**
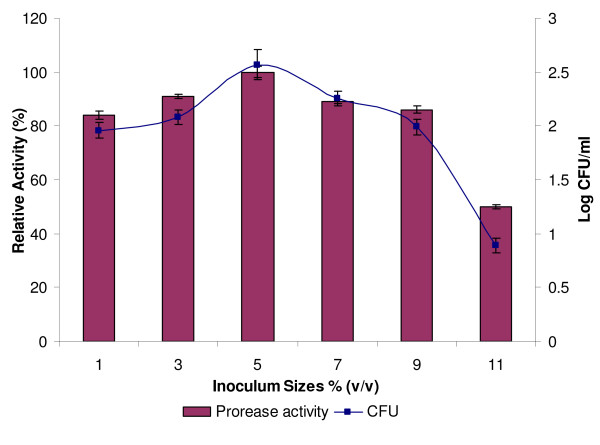
**Effect of inoculum size on protease production**. Culture media were incubated with 1.0, 3.0, 5.0, 7.0, 9.0 and 11.0% (v/v) of inoculum and incubated at 37°C with shaking at 200 rpm for 24 h. Protease activity at with an inoculum size of 5% (v/v) was considered as 100%.

Inoculating several production media, with various inoculum sizes (from 1% to 11%) of the isolate Rand, could affect the production of protease and bacterial growth. The maximum production of protease and bacterial growth were achieved with an inoculum size of 5% (v/v) (Figure 7). Similar result was also reported by Mabrouk *et al*. [32] who found the maximum production of protease by *Bacillus licheniformis *ATCC21415 with an inoculum size of 5% (v/v). A higher inoculum of 11% (v/v) was found to reduce the production of protease more than if the lower inoculum size of 1% (v/v) was used. Therefore, high inoculum sizes do not necessarily give higher protease yield. The increase in the production of protease using small inoculum sizes was suggested to be due to the higher surface area to volume ratio, which resulted in the increased production of protease [14]. In addition, an improved distribution of dissolve oxygen and more effective uptake of nutrient also contributed to a higher protease production. If the inoculum sizes are too small, insufficient number of bacteria would then lead to a reduced amount of secreted protease [33].

However, higher inoculum sizes could lead to or cause a lack of oxygen and depletion of nutrient in the culture media. Different optimum inoculum sizes have been reported by other researchers for different bacteria: 1.0% (v/v) for *Aspergillus flavus *[34] and 4.0% (v/v) for *Pseudomonas aeruginosa *strain K [14].

Under optimized conditions (growth temperature of 37°C; bacterial inoculum size at AB_600 _= 0.5, 5% (v/v); initial pH of 7.0; 24 h of incubated time; agitation rate of 200 rpm), the highest protease activity of 444.7 U/ml (4042.4 U/mg) was obtained.

## Conclusion

The aim of this study was to optimize the physical factors affecting the productions of thermostable and organic solvent-tolerant protease. For this purpose, the organic solvent-tolerant and thermostabe protease were isolated from a newly isolated bacterium (*Bacillus subtilis *strain Rand). The bacterium was identified, based on the 16S rDNA analysis, biochemical tests and morphological study conducted.

It can be concluded that the maximum bacterial growth and production of protease were achieved under optimized conditions.

The extra-cellular protease was found to exhibit a remarkable stability towards several organic solvents. In more specific, it was found to retain 100% and 80% activity at 55 and 60°C, respectively, after 30 min of incubation.

## Methods

### Bacterial isolation

The bacteria used in this study were isolated from contaminated soil mixed with engine oil collected from a car service workshop in Port Dickson, Malaysia. A soil sample (3 g) was suspended in a sterilized Tryptic Soy Broth (TSB) (50 ml). The sample was incubation at 50°C (the temperature during sampling) with an agitation rate at 150 rpm for 24 hr.

### Identification of the bacteria

In this study, *Bacillus subtilis *strain Rand was identified based on the 16S rDNA analysis, morphological properties and biochemical characteristics. The 16S rDNA sequence was amplified via the polymerase chain reaction (PCR), using two universal primers, known as forward (5'-GAGTTTGATCCTGGCTCAG-3') and reverse (5'-CGGCTACCTTGTTACGACTT-3'). The 16S rDNA sequence of *Bacillus subtilis *strain Rand was analyzed using the software package MEGA 4 [35]. Prior to gram staining, pure bacterial strain was streaked on the nutrient agar plate and incubated for 24 h at 50°C for the morphological study. An observation of this was then done under a light microscope. The morphological and physiological characteristics were further determined at Deutsche Sammlung Von Mikroorganismen (DSMZ), Germany. The physiological characteristics study included catalase and oxidase test, anaerobic growth, Voges-Proskauer test, growth at 30, 50 and 55°C, growth in medium at pH 5.7, 2%, 5%, 7% and 10% NaCl, fermentation of D-glucose, L-arabinose, D- xylose, D-mannitol, D-fructose, hydrolysis starch, gelatine, casein and Tween 80, use of citrate and propionate, nitrate reduction, indole production, phenylalanine deaminase and arginine dihydrolase test.

### Production media and growth condition

The culture was grown in standard 500 ml blue cap bottle containing 50 ml of production media. The medium consisted of (g/l); CaCl_2_.2H_2_O 0.5, KH_2_PO_4 _0.2, MgSO_4_.7H_2_O 0.5, NaCl 0.1 and 1% peptone [36]. The pH of the media was adjusted to 7.0 before being autoclaved at 121°C for 15 min. The bacterium was grown for 18 h at 37°C on a shaker at 150 rpm. The culture was centrifuged at 10,000 × g for 10 min and the supernatant was used as crude enzyme for further study.

### Protease assay

The protease activity was determined by a slight modification method proposed by Rahman *et al*. [37]. Azocasein (0.5%, 1 ml) was dissolved in 0.1 M Tris-HCl-2 mM CaCl_2 _pH7.0. The reaction was initiated by adding 100 μl of enzyme solution into the azocasein solution and incubated at 50°C for 30 min. An equal volume of 10% (w/v) TCA was added to terminate the reaction, and the mixture was then allowed at room temperature for 30 min, before centrifugation in eppendoft micro-centrifuge at 13,000 × g for 10 min. The supernatant was removed and mixed with an equal volume of 1 N NaOH. The absorbance was read at 450 nm. One unit of protease activity is defined in the assay conditions, giving an increase of 0.001 absorbance unit at 450 nm per minute [38]. As a control, the enzyme was added at the end of the incubation period.

### Protein assay

Protein was measured using the method suggested by Bradford [39], with bovine serum albumin as the standard.

### Organic solvent-stability of crude enzyme

Three millilitre of crude protease was incubated with 1.0 ml of organic solvent at 50°C with a constant shaking at 150 rpm for 30 min. The proteolytic activities were measured at zero time and the after incubation period using the assay method described above. For control, the solvent was replaced by distilled water. The organic solvents chosen in this study were toluene, *n*-tetradecane, *n*-hexadecane, *n*-dodacane, pyridine, *p*-xylene, *n*-hexane, benzene, *n*-decane and butanol.

### Thermostability of crude enzyme

In this research, the effects of temperature on the crude protease stability were studied. The crude enzyme (without any CaCl_2_) was incubated for 30 min at different temperatures (37, 40, 45, 50, 55, 60, 65, 70°C). The treated enzyme was immediately put in ice-bath for 15 min before measuring the activity. The proteolytic activities were measured at zero time and after the incubation period, using the assay method described in the earlier section.

### The effect of temperature on the production of protease

The ability of the *Bacillus subtilis *strain Rand to grow and produce protease, at elevated temperatures (30 to 65°C), was studied. For this purpose, separate cultures were incubated at 30, 37, 40, 45, 50, 55, 60 and 65°C for 24 h, with agitation at 150 rpm.

### The effect of pH on the production of protease

A loop-full of 24h-old single colony of Rand strain was transferred from a fresh Nutrient agar plate into a 1 L blue cap bottle of production medium (pH 7.0). The culture was incubated at 37°C and 150 rpm on the shaker for 48 hours. Then, some samples were taken at 4 h intervals so as to determine the protease activity and the pH level of the culture medium. The effects of the initial medium pH on the growth and production of protease were studied by adjusting the media (with 1 M NaOH or HCl) to different pH levels ranging from 4 to 13. The media were autoclave, cooled and inoculated with an overnight culture of isolate Rand, and incubated at 37°C (optimum temperature for protease production) for 24 h under 150 rpm of agitation rate.

### The effects of agitation rate on the production of protease

The effects of agitation rate on the growth and production of protease were studied by cultivating the bacteria under different agitation rates (0 to 250 rpm). These cultures were incubated at 37°C for 24 h.

### The effects of inoculum size on the production of protease

The effects of bacterial inoculum size (A_600 _= 0.5) on the growth and production of protease were investigated using different inoculum sizes ranging from 1 to 11% (v/v). The cultures were incubated at 37°C for 24 h, under the agitation rate of 200 rpm (i.e. the optimum agitation rate for the production of protease). Each experiment was carried out in triplicates and the results were taken in the means of three independent determinations.

### Statistical analysis

For statistical analysis, a standard deviation for each experimental result was calculated using the Excel Spreadsheets available in the Microsoft Excel.

## Competing interests

The authors declare that they have no competing interests.

## Authors' contributions

All authors have read and approved the final version of the manuscript.
